# Sense of Coherence and Quality of Life in Patients Treated with Antivitamin K Oral Anticoagulants: A Cross-Sectional Study

**DOI:** 10.3390/ijerph18041668

**Published:** 2021-02-09

**Authors:** Ana Anguas-Gracia, Ana Belén Subirón-Valera, Beatriz Rodríguez-Roca, Ángel Gasch-Gallén, Isabel Antón-Solanas, Fernando Urcola-Pardo

**Affiliations:** 1 Department of Physiatry and Nursing, Faculty of Health Sciences, University of Zaragoza, C/Domingo Miral s/n, 50009 Zaragoza, Spain; aanguas@unizar.es (A.A.-G.); subiron@unizar.es (A.B.S.-V.); brodriguez@unizar.es (B.R.-R.); furcola@unizar.es (F.U.-P.); 2 Research Group Safety and Care (GIISA0021), Institute of Research of Aragón, 50009 Zaragoza, Spain; 3 Research Group Water and Environmental Health (B43_20R), University Institute of Research in Environmental Science of Aragón, University of Zaragoza, 50009 Zaragoza, Spain; 4 Research Group Sector III Healthcare (GIIS081), Institute of Research of Aragón, 50009 Zaragoza, Spain; 5 Research Group Nursing Research in Primary Care in Aragón (GENIAPA) (GIIS094), Institute of Research of Aragón, 50009 Zaragoza, Spain

**Keywords:** quality of life, anticoagulants, primary healthcare, self-efficacy, sense of coherence

## Abstract

The aim of this study was to analyze the correlation between the participants’ self-reported quality of life and their sense of coherence in a sample (*n* = 85) of patients on treatment with oral antivitamin K anticoagulants. A cross-sectional design was used. The measurement instruments included a questionnaire on sociodemographic variables, the Spanish version of the Abbreviated World Health Organization Quality of Life questionnaire (WHOQOL-BREF), an oral-anticoagulant-treatment-specific quality-of-life questionnaire, and the sense-of-coherence (SOC) scale. We analyzed the correlations between the participants’ characteristics and the results from the quality-of-life and SOC scales. Age, level of education, employment status, living arrangement, and treatment length were the determinants of the quality of life in people treated with oral anticoagulants. We found a significant association between the four domains of the WHOQOL-BREF questionnaire and general treatment satisfaction (*p* < 0.01); no significant correlations were found between the SOC subscales and the oral-anticoagulant-treatment-specific quality of life in our sample. Women had a worse level of self-management than men. Nursing interventions should be tailored to the needs of the populations on treatment with oral anticoagulants in order to facilitate a higher level of self-management.

## 1. Introduction

Antivitamin K oral anticoagulants (AKOA) function by reducing plasma concentrations of the vitamin-K-dependent proteins (FII, FVII, FIX, FX, protein C, and protein S) and are crucial in the pharmacological treatment of cardiac and thromboembolic disorders, as well as atrial fibrillation [1]. In Spain, the most frequently prescribed AKOA are warfarin and acenocoumarol. The prevalence of the use of AKOA in Spain is 1.3%, with no significant differences between men and women [2]. Patients on treatment with these AKOA are generally older adults, with an average age of 76 (SD 11.4) [3].

Almost three-quarters of this population (72%) are managed in the community by primary healthcare professionals. This requires frequent follow-up visits in order to guarantee clinical safety through dose adjustment and close monitoring of patients’ sensitivity to treatment [4]. For instance, concomitant treatment with AKOA and antiplatelets such as nintedanib may result in side effects, including gastrointestinal bleeding and perforation [5]. In these cases, individual assessments of bleeding risks involving therapeutic drug monitoring should be carried out regularly. However, the effectiveness of treatment with AKOA is also influenced by other factors such as treatment adherence and disease-coping strategies. Health education is an important tool in the management of patients on treatment with AKOA and should include information related to dietary, absorptive, compliance, and pharmacological factors [6]. Furthermore, a correlation has been found between optimal AKOA treatment control and patients’ illness perception; that is, patients who accept their illness and understand the treatment is more likely to achieve better international normalized ratio (INR) control [7]. However, disease chronicity, impact of the disease on daily life, and the presence of other comorbidities can lead to a reduced quality of life (QoL) in people treated with AKOA, as well as their closest relatives and caregivers [8].

According to the World Health Organization (WHO), the QoL is defined as “individuals’ perception of their position in life in the context of the culture and value systems in which they live and in relation to their goals, expectations, standards and concerns” [9]. Appraising a patient’s QoL allows healthcare professionals to identify health dimensions at risk and also to design individualized interventions to maintain and/or improve the patient/caregiver QoL during treatment with AKOA [10,11]. In addition, according to Antonovsky [12], a self-efficacy approach to health can lead to an improvement in the patients’ understanding, self-management, and meaningfulness of their life, thus generating a higher level of health. According to Antonovsky’s salutogenic model [12], appropriate use of the resources available from the environment provides opportunities for patients to face challenges and recover from adversity; the ability to make appropriate use of these resources and, subsequently, act constructively through life events is known as the sense of coherence (SOC) [13]. A good SOC has been found to be positively related to better health outcomes across a range of patient populations, including patients with cardiac disorders [14,15].

Previous studies have identified a significant association between the QoL and SOC in a range of chronic pathologies, namely cancer [16]; end-stage renal disease [17]; and cardiovascular disorders, including patients undergoing angioplasty procedures [18] and those with chronic heart failure [19], atrial fibrillation [20], and congenital heart disease [21]. A positive correlation between the QoL and SOC has also been established in community-dwelling older people [22,23]. However, less is known about the relationship between the general and specific QoL and SOC, considering the specific QoL as a measurement of QoL in patients on long-term treatment with AKOA. Therefore, the aim of this study was to analyze the relationship between the general and specific QoL and SOC in patients on long-term treatment with AKOA.

## 2. Materials and Methods

### 2.1. Design

A cross-sectional design was used to investigate the correlation between the general and specific QoL and SOC in a sample of patients on treatment with antivitamin KOA.

### 2.2. Participants and Study Location

This study was carried out in a primary care center in the Zaragoza City Center, Spain, which serves a population of 32,862 adult inhabitants (16,659 men and 20,557 women).

The target population comprised all the patients on treatment with AKOA (*n* = 244) who met the selection criteria. The sample size was determined following Mira Tamayo’s recommendations for the application of the Spanish version of the WHO quality-of-life scale (WHOQOL-BREF) [24], resulting in a minimum sample of 75 participants.

A total of 85 patients on treatment with AKOA were recruited to participate in this study. The inclusion criteria to take part in this investigation were (1) community-dwelling adults (aged 18 or over), (2) on treatment with AKOA, (3) managed at the study location, (4) and adherent to treatment as measured by the Morisky Medication Adherence Scale in its Spanish version [25]. We excluded patients who had commenced treatment with AKOA less than 90 days before data collection, those who had difficulty communicating in Spanish, and those who refused to give informed consent. We did not exclude any patients based on the number of comorbidities.

### 2.3. Instruments of Data Collection

We designed an ad hoc questionnaire on sociodemographic and clinical characteristics, including the following variables: sex, age, level of education, employment status, marital status, living arrangement, participation in housework activities, and time since treatment with AKOA.

We used the Spanish version of the WHOQOL-BREF to measure the general QoL in our sample [26]. This scale comprises 24 items classified into four domains, namely physical, psychological, social relationships, and environmental QoL, and two questions to measure the respondents’ self-perceptions of their QoL (item 1) and of their general health (item 2). It is measured on a 5-point Likert scale, with higher scores indicating a higher self-perception of the QoL.

The OA-treatment-specific QoL questionnaire was originally developed by Sawicki [27] in 1999 and was translated and validated for use in a Spanish population by Sánchez González et al. [28] in 2004. This tool measures the QoL in a population on treatment with OA and comprises 32 items divided into five dimensions: (1) treatment satisfaction, (2) self-management, (3) psychological distress, (4) limitations in daily activities, and (5) impact on social activities. The OA-treatment-specific QoL questionnaire is self-administered and is measured on a 6-point Likert scale, with response options ranging from 1 (total disagreement) to 6 (total agreement). Higher mean scores indicate a better QoL in dimensions 1 and 2, and lower mean scores indicate a worse QoL in dimensions 3, 4, and 5. This questionnaire showed good internal consistency for the total scale (α = 0.82) and for each dimension separately [28].

The SOC scale is a self-assessment questionnaire originally developed by Antonovsky [29] to measure the global orientation of the personality, which facilitates an adaptive response to daily-life problems and stressful situations. In this study, we used the Spanish version of the SOC scale validated by Virués-Ortega et al. [30] in 2007. It consists of 13 items classified into three dimensions: (1) comprehensibility (5 items), (2) manageability (4 items), and (3) meaningfulness (4 items). This questionnaire is self-administered, and it is measured on a 7-point Likert scale ranging from 1 (never or rarely) to 7 (very often or always).

### 2.4. Data Analysis

A descriptive analysis was conducted using the mean and standard deviation (SD) for the quantitative variables and frequencies and percentages for the qualitative variables. The differences between groups were analyzed using Student’s *t*-test and Mann–Whitney U test, applying the Bonferroni correction for multiple comparisons. A correlation analysis of the measurement instruments, using the Pearson correlation coefficient, was applied. Finally, stepwise multiple regressions were performed in order to determine the relationship between the significant predictor values for each WHOQOL-BREF dimension. Data codification, processing, and analysis were completed using the software Statistical Package for the Social Science (SPSS) version 22 for Windows (IBM Corp., Chicago, IL, USA), accepting a level of significance of *p* < 0.05.

### 2.5. Ethical Considerations

The investigation adhered to the principles outlined in the Declaration of Helsinki [31]. All the participants included in the study were informed about the study’s aims and procedures and gave their informed consent to participate. The research protocol was authorized by the directive managers of Aragón’s Primary Care Department and was approved by the Clinical Research Ethics Committee of Aragón (C.P.—C.I. PI18/0177).

## 3. Results

A total of 85 patients completed the questionnaires. The sociodemographic characteristics of our sample are described in Table 1. The majority of our participants were men (60%), with a mean age of 76.24 years (SD: 10.7 years). Nearly half of our participants were educated to the primary school level; the remaining participants were educated to the secondary school or university level, and only five of our patients said that they had not studied. Most of our patients were retired (90.6%), and just over half of our sample were married. With regard to their living arrangements, 22.4% of our patients lived alone, 43.5% lived with their partners, and 31.8% lived with relatives other than their partners. Almost half (47.1%) of our participants said that they contributed to housework tasks. The mean number of months on treatment with AKOA was 74.08 (SD: 62.53).

The associations between the sociodemographic characteristics of our participants and the results from the WHOQOL-BREF questionnaire are presented in Table 2. We observed that the patients aged less than 80 years and those who were educated to at least the secondary school level presented a higher QoL in the physical domain (*p* < 0.01). A higher QoL in the psychological domain was observed in patients aged less than 80 years old (*p* < 0.01), married patients (*p* < 0.05), and those who lived with their partners (*p* < 0.01). People who were unemployed reported a lower QoL in the social domain (*p* < 0.05). In the environmental domain, the participants who were married (*p* < 0.05), those who did not perform any housework tasks (*p* < 0.05), and those who had been on treatment for less than five years presented a higher QoL (*p* < 0.05). It is important to note that after applying the Bonferroni correction, only the association between age and the QoL in the psychological domain remained significant.

The results from the OA-treatment-specific QoL questionnaire are presented in Table 3. We observed significant differences in the self-management domain, with better results for the men (*p* < 0.01), the participants who were educated to the secondary school level or above (*p* < 0.05), and those who were married (*p* < 0.05). The patients who lived with their partners, as opposed to those living alone or with relatives other than their partners, experienced fewer limitations in daily activities (*p* < 0.05). We did not find any significant differences between groups for the treatment satisfaction, impact on social activities, and psychological distress domains. After the Bonferroni correction, none of the associations remained significant.

Table 4 shows the results from the SOC scale. We found a significant association between the length of treatment with AKOA (less than five years) and the manageability (*p* < 0.01) and meaningfulness dimensions (*p* < 0.05). We did not identify any additional significant differences between groups in the manageability dimension. However, we observed that the patients who were aged 80 years or less achieved better results in the meaningfulness dimension (*p* < 0.01). Significant differences were observed in the comprehensibility dimension, with married patients (*p* < 0.05), those who lived with their partners (*p* < 0.01), and those who did not take part in housework tasks (*p* < 0.01) achieving better results. All of the associations remained significant with the exception of marital status–comprehensibility and treatment length–meaningfulness after the Bonferroni correction.

We analyzed the correlations between the three questionnaires (Table 5). The four domains of the WHOQOL-BREF were associated with the OA-treatment-specific QoL questionnaire dimension treatment satisfaction. We did not find any significant associations between the WHOQOL-BREF and the SOC dimensions. We identified a positive correlation between the self-management domain from the OA-treatment-specific QoL questionnaire and the psychological domain from the WHOQOL-BREF questionnaire; self-management was also positively correlated with the comprehensibility dimension from the SOC scale. With regard to the impact on social activities, as measured by the OA-treatment-specific QoL questionnaire, we identified significant inverse correlations with the physical, psychological, and social WHOQOL-BREF domains and with the manageability and comprehensibility SOC subscales. Limitations in daily activities and psychological distress were inversely correlated with the four domains of the WHOQOL-BREF questionnaire and the three SOC subscales. We observed a significant positive association between the WHOQOL-BREF’s physical, psychological, and environmental domains and the SOC scale.

Multivariate regression was performed to predict the values of each WHOQOL-BREF dimension (Table 6). For the physical domain, the significant equation was predicted by OA-treatment-specific QoL-General Treatment Satisfaction (GTS), SOC-Comp, level of education, SOC-Meaning, and SOC-Man (*r*^2^ = 0.49), while the psychological domain equation was based on SOC-Comp, OA-treatment-specific QoL-Distress (OAQoL-D), and age (*r*^2^ = 0.54). For the social domain, the significant equation was based on OAQoL-D and employment (*r*^2^ = 0.25), and the predictor model for the environmental dimension was based on the values of SOC-Meaning, SOC-Comp, and OAQoL-GTS (*r*^2^ = 0.30).

## 4. Discussion

This study analyzed the relationship between the general and specific QoL in a population of patients on treatment with AKOA and their SOC. In addition, we analyzed the associations between the patients’ sociodemographic characteristics and the results from the questionnaires used.

An association was established between certain patient characteristics and a better physical, psychological, and environmental QoL, particularly being younger, having a higher level of education, living with a partner, being employed, and having a shorter AKOA treatment time. Our results are consistent with those reported by Gobbens and Remmen [32] in a previous study. In addition, our findings are consistent with those obtained by Criado-Álvarez et al. [33] in a similar study in the Spanish population treated with OA. Although no statistical differences between men and women were found, women’s social QoL was comparatively lower than men’s. This was also observed by Ko et al. [34] in a study in South Korea. This may be explained by a number of reasons, including the fact that women have a higher life expectancy and, thus, are at risk of having comorbidities, experiencing chronic pain and functional and/or cognitive impairments [35].

We identified self-management and gender as key determinants of the level of the OA-treatment-specific QoL in our sample. This was also observed in similar studies with different populations [36]. This trend may be suggestive of the need for nurses to design and implement alternative health promotion interventions in order to improve self-management of AKOA treatment. Moreover, it was interesting to observe that the results from the OA-treatment-specific QoL measure were different from those yielded by a general QoL measure. Therefore, it may be advisable for healthcare professionals to complement general QoL assessments with disease- or treatment-specific tools.

Previous studies [37] have identified the SOC as an effective contributor to a better QoL. In older adult populations, high SOC scores have been related to lower mortality rates and less functional decline, independently of multimorbidity, depression, cognition, disability, and sociodemographic characteristics [38]. A previous study [30] carried out in Spain showed that a high SOC protected against stress-related disorders and the development of unhealthy behaviors. In addition, the perception of controllability had protective physiological effects in patients with a chronic health condition [21,30].

There are few studies addressing the SOC in patients on treatment with AKOA. However, according to our results, there may be an association between the SOC and QoL in this population. In particular, manageability, which refers to a person’s own control resources from an instrumental/behavioral point of view [39], was associated with shorter time on treatment with AKOA. This may suggest that follow-up programs and long-term interventions are necessary to reinforce the skills and motivations acquired in the first few months of treatment [40]. Similarly, meaningfulness was associated in our population with a higher level of education, which, in turn, was predictive of the OA-treatment-specific QoL dimension self-management [41]. Meaningfulness is known as the motivational component of the SOC and allows people to see challenges where others only see burdens [39]. Our younger participants achieved a higher level of meaningfulness, which may point at a need to adapt self-management educational programs to the needs and characteristics of the different age groups [42]. Comprehensibility is defined as the cognitive SOC dimension and facilitates the management of information [39]. In our sample, a higher level of comprehensibility was correlated to being married, living with one’s partner, and not participating in housework tasks. Although more evidence is needed to support this argument, we suggest that patients on treatment with AKOA may benefit from a distraction-free environment that allows them to focus on the information given. However, (the lack of) participation in housework tasks may not be a matter of choice for some of our patients, resulting in health inequalities and worse disease management for some of our participants [43,44].

We found that OA-treatment-specific QoL scores were associated with better internal resources, as measured by the SOC scale [45]. In our study, the self-management domain was correlated with the results from the SOC’s comprehensibility subscale. This supports our previous suggestion that it is important to adapt health education and health promotion interventions to the needs and characteristics of the population on treatment with AKOA in order to improve self-management [42]. We also found a correlation between the WHOQOL-BREF’s physical, psychological, and environmental domains and the patients’ SOC. This is in agreement with a previous study [46] analyzing the relationship between the SOC and research, which identified a significant association between the SOC and QoL in a sample of older adults with severe chronic heart failure.

Our results suggest that closer attention should be paid to the patients’ ability to self-manage their AKOA treatment. Their ability to self-manage their treatment, and thus their condition, has an impact on their social life and their activities for daily living, which, in turn, are also affected by other factors, such as their living arrangements and their level of psychological distress. Individually tailored interventions are essential to improve the patients’ ability to confront daily challenges [47]. In addition, the need to involve the patients’ partners in health education and promotion interventions should be considered [8]. Finally, a higher SOC is independently associated with age, sex, and education and results in better health outcomes and healthier lifestyle choices. Therefore, individual differences in the patients’ SOC should be taken into account when designing and implementing health education and promotion interventions [48].

This study had some limitations that need to be acknowledged. First, the methodology employed in this investigation did allow for the establishment of associations between variables but not causality. Second, our sample was representative of the patients registered at a primary care center in the city of Zaragoza; however, the generalizability of our results to the general population of Spanish patients on treatment with antivitamin KOA is limited and thus our results should be interpreted with caution. Finally, differences in the patients’ QoL were found between the generic QoL scale and the OA-treatment-specific quality-of-life questionnaire. This may be explained by the impact of external variables, such as the number of comorbidities, that were not measured as part of this investigation. Therefore, we suggest that future research in this area includes measurement of clinical variables relating to the patients’ general health status.

## 5. Conclusions

Patients who were younger, had a higher level of education, were in employment, and were living with their partners generally had a better QoL. However, differences were found in the participants’ general and OA-treatment-specific quality of life. Specifically, although after the Bonferroni correction, the strength of the association was diminished, being a man, being married, and having a higher level of education were indicators of better levels of a treatment-specific QoL. This suggests that assessment of the QoL in patients on treatment with AKOA should include both general and treatment-specific measures. In addition, our data indicate that the patients’ level of meaningfulness, comprehensibility, and manageability have an impact on their general and treatment-specific QoL. This suggests that both specific characteristics and individual differences in the patients’ SOC should be considered during follow-ups and monitoring.

## Figures and Tables

**Table 1:** Sociodemographic characteristics (*n* = 85).

Variable	Category	*n* (%)
Sex	Male	51 (60%)
	Female	34 (40%)
Age	Less than 80 years	44 (51.8%)
	80 years or more	41 (48.2%)
Level of education	Primary school or below	47 (55.3%)
	Secondary school or above	38 (44.7%)
Employment status	Retired	77 (90.6%)
	Employed	8 (9.4%)
Marital status	Married	45 (52.9%)
	Single or widowed	40 (47.1%)
Living arrangement	Living with partner	37 (43.5%)
	Living alone or with relatives other than partner	48 (56.5%)
Participation in housework	Yes	40 (47.1%)
	No	45 (52.9%)
Length of treatment with AKOA	Less than 5 years	43 (50.6%)
	5 years or more	42 (49.4%)

**Table 2:** Associations between the participants’ sociodemographic characteristics and the results from the World Health Organization quality-of-life scale (WHOQOL-BREF) questionnaire.

Variable	Category	*n* (%)	WQ-Ph				WQ-Psy				WQ-Social				WQ-Env			
			Mean (SD)	df	*p*	*p* ^B^	Mean (SD)	df	*p*	*p* ^B^	Mean (SD)	df	*p* ^B^	Corrected	Mean (SD)	df	*p*	*p* ^B^
Sex ^1^	Male	51 (60%)	13.17 (2.08)	83	0.08	0.48	14.56 (1.78)	83	0.14	0.70	13.15 (2.07)	83	0.15	0.72	14.73 (1.66)	83	0.71	0.99
	Female	34 (40%)	12.18 (3.01)				13.88 (2.51)				13.88 (2.55)				14.59 (1.77)			
Age ^1^	Less than 80 years	44 (51.8%)	13.36 (2.58)	83	0.025	0.18	14.89 (1.75)	83	0.006	0.047	13.73 (2.38)	83	0.23	0.87	14.86 (1.68)	83	0.28	0.93
	80 years more	41 (48.2%)	12.14 (2.33)				13.64 (2.29)				13.14 (2.16)				14.46 (1.70)			
Level of education ^1^	Primary school or below	47 (55.3%)	12.16 (2.76)	83	0.009	0.07	14.13 (2.34)	83	0.43	0.99	13.70 (2.35)	83	0.25	0.89	14.65 (1.81)	83	0.89	1
	Secondary school or above	38 (44.7%)	13.53 (1.98)				14.49 (1.80)				13.12(2.18)				14.70 (1.57)			
Employment ^2^	Retired	77 (9.4%)	12.55 (2.37)	-	0.08	0.49	14.18 (2.05)	-	0.28	0.92	13.23 (2.13)	-	0.029	0.20	14.58 (1.58)	-	0.22	0.86
	Employed	8 (90.6%)	14.93 (3.14)				15.33 (2.52)				15.50 (2.85)				15.56 (2.53)			
Marital status ^1^	Married	45 (52.9%)	13.17 (2.16)	83	0.13	0.67	14.80 (1.86)	83	0.017	0.13	13.36 (2.00)	83	0.74	1	15.04 (1.49)	83	0.030	0.21
	Single or widowed	40 (47.1%)	12.33 (2.85)				13.72 (2.25)				13.53 (2.59)				14.25 (1.83)			
Living arrangement ^1^	Living with partner	37 (43.5%)	13.24 (2.22)	83	0.13	0.67	14.97 (1.77)	83	0.008	0.06	13.51 (1.89)	83	0.79	1	15.05 (1.49)	83	0.06	0.39
	Not living with partner	48 (56.5%)	12.42 (2.70)				13.76 (2.22)				13.39 (2.56)				14.28 (1.80)			
Participation in homework activities ^1^	Yes	40 (47.1%)	12.54 (2.29)	83	0.43	0.98	13.92 (1.78)	83	0.12	0.64	13.00 (2.33)	83	0.09	0.52	14.28 (1.64)	83	0.042	0.29
	No	45 (52.9%)	12.98 (2.73)				14.62 (2.34)				13.84 (2.19)				15.02 (1.68)			
Treatment length ^1^	Less than 5 years	43 (50.6%)	13.18 (2.80)	83	0.13	0.67	14.70 (1.94)	83	0.07	0.44	13.83 (2.54)	83	0.11	0.61	15.07 (1.75)	83	0.027	0.19
	5 years or more	42 (49.4%)	12.35 (2.16)				13.87 (2.22)				13.05 (1.94)				14.26 (1.55)			
Total		85 (100%)	12.77 (2.52)	-			14.29 (2.11)	-			13.44 (2.28)	-			14.67 (1.69)	-		

^1^ Student’s *t*-test; ^2^ Mann–Whitney’s U test; WQ-Ph: physical domain; WQ-Psy: psychological domain; WQ-Social: social domain; WQ-Env: environmental domain; *p*: raw *p*-value; *p*^B^: *p*-value after the Bonferroni correction.

**Table 3:** Associations between the participants’ sociodemographic characteristics and the results from the oral anticoagulant (OA)-treatment-specific QoL questionnaire.

Variable	Category	*n* (%)	Treatment Satisfaction				Self-Management				Psychological Distress				Limitations in Daily Activities				Impact on Social Activities			
			Mean (SD)	df	*p*	*p* ^B^	Mean (SD)	df	*p*	*p* ^B^	Mean (SD)	df	*p*	*p* ^B^	Mean (SD)	df	*p*	*p* ^B^	Mean (SD)	df	*p*	*p* ^B^
Sex ^1^	Male	51 (60%)	4.79 (0.76)	83	0.25	0.89	4.71 (0.61)	83	0.009	0.069	1.96 (0.66)	83	0.94	1	2.09 (0.67)	83	0.12	0.64	3.00 (0.66)	83	0.10	0.57
	Female	34 (40%)	4.57 (0.96)				4.26 (0.93)				1.97 (0.90)				2.36 (0.92)				3.30 (0.99)			
Age ^1^	Less than 80 years	44 (51.8%)	4.77 (0.86)	83	0.42	0.98	4.48 (0.88)	83	0.55	0.99	2.02 (0.75)	83	0.48	0.99	2.26 (0.79)	83	0.44	0.99	3.11 (0.75)	83	0.93	1
	80 years or more	41 (48.2%)	4.63 (0.82)				4.58 (0.66)				1.90 (0.77)				2.13 (0.78)				3.13 (0.88)			
Level of education ^1^	Primary school or below	47 (55.3%)	4.70 (0.87)	83	0.99	1	4.36 (0.92)	83	0.029	0.21	2.04 (0.86)	83	0.28	0.92	2.10 (0.72)	83	0.21	0.85	3.04 (0.85)	83	0.32	0.95
	Secondary school or above	38 (44.7%)	4.70 (0.82)				4.73 (0.51)				1.87 (0.61)				2.32 (0.85)				3.22 (0.76)			
Employment ^2^	Retired	77 (9.4%)	4.71 (0.82)	-	0.87	1	4.49 (0.80)	-	0.15	0.72	1.99 (0.78)	-	0.39	0.98	2.18 (0.75)	-	0.60	0.99	3.08 (0.82)	-	0.26	0.91
	Employed	8 (90.6%)	4.60 (1.14)				4.91 (0.40)				1.70 (0.49)				2.43 (1.09)				3.47 (0.73)			
Marital status ^1^	Married	45 (52.9%)	4.75 (0.73)	83	0.57	0.99	4.68 (0.58)	83	0.049	0.33	1.97 (0.62)	83	0.93	1	2.10 (0.78)	83	0.19	0.81	3.02 (0.74)	83	0.23	0.88
	Single or widowed	40 (47.1%)	4.65 (0.96)				4.35 (0.93)				1.96 (0.90)				2.32 (0.78)				3.24 (0.89)			
Living arrangement ^1^	Living with partner	37 (43.5%)	4.83 (0.72)	83	0.19	0.81	4.67 (0.61)	83	0.12	0.64	1.95 (0.60)	83	0.90	1	1.98 (0.63)	83	0.020	0.14	2.95 (0.71)	83	0.09	0.53
	Not living with partner	48 (56.5%)	4.60 (0.92)				4.42 (0.88)				1.97 (0.87)				2.37 (0.85)				3.25 (0.87)			
Participation in housework tasks ^1^	Yes	40 (47.1%)	4.63 (0.95)	83	0.49	0.99	4.38 (0.91)	83	0.10	0.57	2.09 (0.90)	83	0.14	0.70	2.36 (0.82)	83	0.07	0.44	3.19 (0.88)	83	0.49	0.99
	No	45 (52.9%)	4.76 (0.74)				4.66 (0.62)				1.84 (0.58)				2.05 (0.73)				3.06 (0.76)			
Treatment length ^1^	Less than 5 years	43 (50.6%)	4.74 (0.90)	83	0.64	0.99	4.42 (0.92)	83	0.20	0.83	1.86 (0.64)	83	0.19	0.81	2.12 (0.78)	83	0.36	0.97	3.09 (0.82)	83	0.69	0.99
	5 years or more	42 (49.4%)	4.66 (0.79)				4.64 (0.60)				2.07 (0.85)				2.28 (0.78)				3.16 (0.81)			
Total		85 (100%)	4.70 (0.84)	-			4.53 (0.78)	-			1.96 (0.76)	-			2.20 (0.78)	-			3.12 (0.81)	-		

^1^ Student’s *t*-test; ^2^ Mann–Whitney’s U test; *p*: raw *p*-value; *p*^B^: *p*-value after the Bonferroni correction.

**Table 4:** Associations between the participants’ sociodemographic characteristics and the results from the sense-of-coherence (SOC) scale.

Variable	Category	*n* (%)	SOC-Manageability				SOC-Comprehensibility				SOC-Meaningfulness			
			Mean (SD)	df	*p*	*p* ^B^	Mean (SD)	df	*p*	*p* ^B^	Mean (SD)	df	*p*	*p* ^B^
Sex ^1^	Male	51 (60%)	5.14 (0.86)	83	0.79	1	5.08 (0.90)	83	0.06	0.39	5.09 (0.83)	83	1	1
	Female	34 (40%)	5.08 (1.16)				4.67 (1.05)				5.09 (1.93)			
Age ^1^	Less than 80 years	44 (51.8%)	5.12 (0.88)	83	0.96	1	4.84 (1.06)	83	0.45	0.99	5.37 (0.88)	83	0.003	0.024
	80 years or more	41 (48.2%)	5.11 (1.09)				5.00 (0.89)				4.79 (0.85)			
Level of education ^1^	Primary school or below	47 (55.3%)	5.15 (1.10)	83	0.71	0.99	4.91 (1.11)	83	0.98	1	4.94 (0.90)	83	0.09	0.53
	Secondary school or above	38 (44.7%)	5.07 (0.82)				4.92 (0.80)				5.27 (0.90)			
Employment ^2^	Retired	77 (9.4%)	5.13 (1.00)	-	0.59	0.99	4.92 (0.97)	-	0.99	1	5.05 (0.87)	-	0.11	0.61
	Employed	8 (90.6%)	5.00 (0.83)				4.88 (1.10)				5.50 (1.23)			
Marital status ^1^	Married	45 (52.9%)	5.19 (0.94)	83	0.43	0.98	5.16 (0.80)	83	0.014	0.11	5.24 (0.74)	83	0.11	0.61
	Single or widowed	40 (47.1%)	5.03 (1.03)				4.65 (1.09)				4.92 (1.05)			
Living arrangement ^1^	Living with partner	37 (43.5%)	5.22 (0.99)	83	0.38	0.97	5.26 (0.76)	83	0.003	0.024	5.07 (0.68)	83	0.89	1
	Not living with partner	48 (56.5%)	5.03 (0.98)				4.65 (1.05)				5.10 (1.06)			
Participation in housework tasks ^1^	Yes	40 (47.1%)	4.99 (0.93)	83	0.29	0.93	4.61 (1.06)	83	0.005	0.039	5.19 (0.84)	83	0.34	0.96
	No	45 (52.9%)	5.22 (1.02)				5.20 (0.81)				5.00 (0.97)			
Treatment length ^1^	Less than 5 years	43 (50.6%)	5.40 (1.03)	83	0.006	0.047	5.07 (1.06)	83	0.15	0.73	5.31 (0.91)	83	0.023	0.17
	5 years or more	42 (49.4%)	4.82 (0.85)				4.76 (0.87)				4.86 (0.86)			
Total		85 (100%)	5.11 (0.98)	-			4.92 (0.98)	-			5.09 (0.91)	-		

^1^ Student’s *t*-test; ^2^ Mann–Whitney’s U test; *p*: raw *p*-value; *p*^B^: *p*-value after the Bonferroni correction.

**Table 5:** Pearson’s correlations between the assessed dimensions.

	WQ-Ph	WQ-Psy	WQ-Social	WQ-Env	OAQoL-GTS	OAQoL-SE	OAQoL-SSN	OAQoL-DH	OAQoL-D	SOC-Man	SO-Comp	SO-Meaning
WQ-Ph	-											
WQ-Psy	0.601 **	-										
WQ-Social	0.429 **	0.500 **	-									
WQ-Env	0.575 **	0.634 **	0.534 **	-								
OAQoL-GTS	0.453 **	0.299 **	0.356 **	0.303 **	-							
OAQoL-SE	0.263 *	0.176	0.043	0.143	0.025	-						
OAQoL-SSN	−0.349 **	−0.231 *	−0.253 *	−0.184	−0.535 **	−0.090	-					
OAQoL-DH	−0.322 **	−0.341 **	−0.314 **	−0.261 *	−0.647 **	−0.161	0.660 **	-				
OAQoL-D	−0.395 **	−0.509 **	−0.363 **	−0.322 **	−0.571 **	0.059	0.557 **	0.688 **	-			
SOC-Man	0.232 *	0.458 **	0.187	0.257 *	0.178	0.057	−0.405 **	−0.398 **	−0.384 **	-		
SOC-Comp	0.436 **	0.524 **	0.253 *	0.383 **	0.126	0.357 **	−0.256 *	−0.364 **	−0.249 *	0.663 **	-	
SOC-Meaning	0.373 **	0.431 **	0.185	0.392 **	0.087	−0.041	−0.076	−0.072	−0.253 *	0.340 **	0.251 *	-

WQ-Ph: physical domain; WQ-Psy: psychological domain; WQ-Social: social domain; WQ-Env: environmental domain; OAQoL-GTS: general treatment satisfaction; OAQoL-SE: self-efficacy; OAQoL-SSN: strained social network; OAQoL-DH: daily hassles; OAQoL-D: distress; SOC-Man: manageability; SOC-Comp: comprehensibility; SOC-Meaning: meaningfulness; * *p* < 0.05; ** *p* < 0.01.

**Table 6:** Regression analysis for the WHOQOL-BREF dimensions.

Dependent Variable	Independent Variables Included	*B* (Std. Error)	*β*	DF-Reg ^a^	DF-Res ^b^	*F*	*r* ^2^	*p*	D-W ^c^	Excluded Variables
WQ-Ph	OAQoL-GTS	1.24 (0.24)	0.41	79	5	15.49	0.49	0.000	1.92	Age, OAQoL-SE, OAQoL-SSN, OAQoL-DH, OAQoL-D
	SOC-Comp	1.23 (0.28)	0.48							
	Level of education	1.09 (0.42)	0.21							
	SOC-Meaning	0.72 (0.24)	0.26							
	SOC-Man	0.61 (0.29)	0.24							
WQ-Psy	SOC-Comp	0.98 (0.17)	0.45	81	3	31.21	0.54	0.000	2.18	OAQoL-GTS, OAQoL-SE, OAQoL-SSN, OAQoL-DH, SOC-Man, SOC-Meaning, marital status, living arrangement
	OAQoL-D	−1.02 (0.20)	−0.39							
	Age	−1.39 (0.32)	−0.33							
WQ-Social	OAQoL-D	−1.16 (0.27)	−0.41	82	2	13.83	0.25	0.000	2.15	OAQoL-GTS, OAQoL-SSN, OAQoL-DH, SOC-Comp
	Employment	−2.72 (0.75)	−0.35							
WQ-Env	SOC-Meaning	0.56 (0.18)	0.30	81	3	11.44	0.30	0.000	2.39	Marital status, participation in homework activities, treatment length, OAQoL-DH, OAQoL-D, SOC-Man
	SOC-Comp	0.48 (0.17)	0.28							
	OAQoL-GTS	0.49 (0.19)	0.24							

WQ-Ph: physical domain; WQ-Psy: psychological domain; WQ-Social: social domain; WQ-Env: environmental domain; OAQoL-GTS: general treatment satisfaction; OAQoL-SE: self-efficacy; OAQoL-SSN: strained social network; OAQoL-DH: daily hassles; OAQoL-D: distress; SOC-Man: manageability; SOC-Comp: comprehensibility; SOC-Meaning: meaningfulness; ^a^ DF-Reg: regression degrees of freedom; ^b^ DF-Res: residual degrees of freedom; ^c^ D-W: Durbin–Watson test.

## Data Availability

The data presented in this study are available on request from the corresponding author. The data are not publicly available due to specific requirements from the clinical research ethics committee that reviewed and approved this investigation.
